# Maintenance vs. Change of Negative Therapy Expectation: An Experimental Investigation Using Video Samples

**DOI:** 10.3389/fpsyt.2022.836227

**Published:** 2022-04-04

**Authors:** Kristina Braun-Koch, Winfried Rief

**Affiliations:** Department of Clinical Psychology and Psychotherapy, Philipps-University Marburg, Marburg, Germany

**Keywords:** therapy expectation, attitudes towards psychotherapy, ViolEx model, expectation violation, video intervention

## Abstract

**Introduction:**

Therapy expectations contribute substantially to the outcome of psychotherapy. In contrast, psychotherapy expectations are rarely addressed and systematically optimised in studies on psychotherapy.

**Materials and Methods:**

A total of 142 mostly healthy participants with critical attitudes towards psychotherapy were randomised into two groups: (1) a control group that watched a video with patients who gave information about their symptoms or (2) an experimental group that watched an expectation-optimised video with the same patients giving additional information about their mostly positive therapy outcomes. The primary outcome was the Milwaukee Psychotherapy Expectation Questionnaire (MPEQ), which was filled in before and after watching the video.

**Results:**

Both groups showed a significant improvement of their process expectations and attitudes towards psychotherapy after watching the video. Participants in the experimental group changed their therapy outcome expectation while there was no change in the control group [*F*(1,140) = 9.72, *p* = 0.002, η2 = 0.065].

**Conclusion:**

A video intervention with patients presenting their positive therapy experiences improves therapy expectations in persons with critical attitudes. Expectation-optimised videos could be used for prevention programmes and when starting therapy.

**Trial Registration:**

Trial was registered at clinicaltrials.gov (NCT03594903) on November 2018.

## Introduction

Psychological interventions are effective methods for treating various mental diseases. While the number of people with mental disorders is increasing worldwide, access to appropriate care is still denied to a large proportion. This disparity is all the more tragic because more and more effective treatments are available (1). In a “Study on Adult Health in Germany” (DEGS1-MH) by Jacobi et al. (2), a 12-month prevalence of mental disorders of almost 28% was found. At the same time, the treatment rate was very low: only 11–40% of affected patients had used health services within the past year. Not treating mental disorders leads to serious health, social and economic consequences (2, 3), which makes improving access to psychological treatments crucial.

Barriers to psychotherapeutic treatment can be found at all levels of care (1). Obstacles to care also exist for the patients themselves. On the one hand, in some countries, framework conditions such as time management, travel and searching for therapists represent practical hurdles, beyond the issue of financial compensation. On the other hand, various patient characteristics hinder their ability to find adequate care. These include a lack of motivation, fear of stigmatization, and a low or critical therapy expectation (4, 5). Besides unfavourable utilization rates due to these patient characteristics, another consequence is the premature termination of therapy; this represents one of greatest challenges in ensuring adequate treatment success (6). In addition, patient features such as therapy expectation are generally regarded as factors that in part determine the effectiveness of psychotherapy (7–10). As a consequence the development of effective interventions that promote positive expectations of psychotherapy holds great potential for healthcare. Such interventions could be particularly useful for people with critical attitudes towards psychotherapy because people with negative expectations benefit less from psychotherapy or do not even make use of it (7, 9, 10).

The power of expectation also underlies another known effect: the placebo effect (11). A placebo effect is generally understood to be the positive effect of a chemically ineffective drug (or other inactive treatment). It has been proven in various pharmacological and medical settings and is mainly determined by positive expectations of the treatment outcome (12). While for a long time placebos were used exclusively to find out what proportion of the treatment effect was due to its “true effect” and what proportion to non-specific influences, researchers now demand that the placebo effect should also be used in active interventions in order to maximise treatment success (13). To this end, the authors suggest actively promoting positive expectations of treatment, which applies not only to medical, but also to psychotherapeutic treatments (14, 15). However, in line with the professional ethics of psychotherapists and the negative effect of exaggerated expectations, only realistic expectations of psychotherapy should be promoted (16).

So far, there have been different approaches to improve therapy expectations. Mitchell and Gordon (17) showed that expectations regarding an online-based cognitive behavioural therapy increased significantly as a result of a demonstration of the programme. The effect was primarily based on the reduction of misunderstandings and false assumptions. Another way to positively influence expectations is to address them directly. Various authors recommend discussing discrepancies between expectations of the therapy process (e.g., duration, patients’ and therapists’ roles) and the current therapy process with patients (15, 18). Presenting positive therapy results *a priori* has also proved to be helpful in increasing positive outcome expectations (19, 20). In a study by van Osch et al. (21) the targeted induction of positive treatment expectations played a central role in boosting therapy expectations.

Another way to change treatment expectations is the use of persuasive communication tactics (22). A well-known model that describes the process of persuasion is the elaboration likelihood model (23). The authors postulated that persuasion could take place *via* two routes: the central and the peripheral routes. A conviction achieved on the central route is more resistant and a better predictor of future behaviour (24). Due to this advantage, a conviction on this route–i.e., using rational arguments–is usually also recommended in the literature about expectation change; Constantino et al. (14), for example, recommended addressing knowledge about the effectiveness of treatment and a convincing therapeutic rationale. In fact, in a study by Ahmed and Westra (25), the presentation of a convincing therapeutic rational for the treatment of social phobia proved to be an effective method for raising expectations about treatment.

Social learning can also be seen to be an underlying mechanism of changing therapy expectations. Findings from placebo and nocebo research support this statement. The study by Colloca and Benedetti (26) proved that observational learning can cause a strong placebo reaction, thereby improving outcomes. This was also shown by a study of Kazdin and Krouse (20): participants who had heard audio recordings with positive reports from patients reported higher expectations of treatment success. Consequently, Rief and Glombiewski (18) recommended sharing therapy experiences with other patients or watching videos of success stories of psychotherapeutic treatments as a possible expectation-increasing intervention.

A review by Tinsley et al. (27) determined that, of the various media with which an expectation-changing intervention can be carried out, audio and video material have proven to be superior to personal conversations or written materials. In line with this finding recent studies have chosen video material as a medium to change therapy expectations (21, 28).

To summarise, it can be argued that expectations could be successfully improved through various interventions. The use of video material seems most promising. Important mechanisms by which expectations can be changed seem to be the specific referencing of positive treatment outcomes, the use of persuasive communication (especially the use of rational arguments), and social learning. So far, no study has combined the facets of expectation-changing interventions in one single study.

The following question was addressed in this study: Can expectations about psychotherapy be improved by watching an expectation-optimizing video in a population with critical expectations towards psychotherapy?

As a central hypothesis, the influence of expectation-optimised video on a change in expectations was examined. Although in the intervention video outcome expectations of psychotherapy were specifically addressed, in the control video mainly symptoms and medical histories of psychologically burdened patients were discussed. Based on the Allport contact hypothesis (29), it was expected that even virtual contact with mentally ill people and the discussion of psychotherapy would lead to a reduction in prejudices against mental disorder and their treatment both in the control and intervention groups. However, due to the explicit addressing of positive expectations in the intervention video compared with the control video, it was assumed that the intervention video would lead to a larger increase in positive expectations at T1 compared with T0. Similar patterns of results were expected for secondary outcome variables, such as attitudes towards psychotherapy and openness towards using psychotherapy.

## Materials and Methods

**FIGURE 1:**
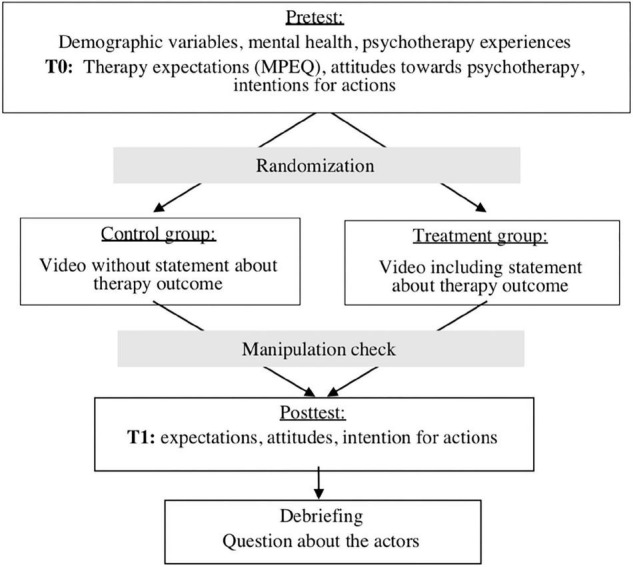
Study design.

An overview of the procedure can be found in Figure 1.

The survey was conducted online using UniPark from November 2018 to January 2019. To recruit the sample, a link to the study was distributed *via* the university’s e-mail distribution list and *via* social media. In addition, flyers were distributed in the city of Marburg. Inclusion criteria were a minimum age of 18 years, good German language skills, and a self-rated critical attitude towards psychotherapy. Exclusion criteria were being a student of psychology or the presence of one or more of the following diagnoses: dementia, addiction, or psychosis. As an incentive, the respondents could win one of four Amazon vouchers worth €50. The desired sample size was determined in an *a priori* power analysis using G*Power 3.1.9.2^®^ [(30), RRID:SCR_013726] for the central hypothesis. The expected effect was estimated at *f* = 0.25, the α level was set at 0.05 and the power at 1–β = 0.90. A sample size of 44 participants was obtained. After consideration of possible dropouts of approximately 10–15%, the desired sample size was 50 participants As the online recruitment worked very well, we exceeded the targeted sample size very quickly. The participants were randomly assigned to one of two conditions using the quota distribution in UniPark.

First, participants were informed about the procedure and content of the study (why and how negative attitudes towards psychotherapy persist). Informed consent, a questionnaire on demographic variables, and information on mental health were collected. This was followed by the first measurement (T0) of therapy expectations and attitudes towards psychotherapy as well as behavioural intentions. Experimental manipulation then took place. Depending on the assigned condition, the participants were shown one of two videos (intervention or control video). Subsequently, a manipulation check was performed to make sure that the participants had watched the video attentively. Then, the second measurement (T1) took place. Finally, the participants were debriefed about the fictitious character of the patients and the manipulation. The total duration of the experiment was 30 min.

### Video Intervention

We asked experts (psychotherapists and scientists in the area of clinical psychology) about typical therapy expectation violations in therapy (from negative to positive expectations) and searched the literature for information about typical therapy processes and outcomes. Based on this information, we designed a script for the experimental video (see Supplementary Material). The patients in the video were played by actors aged from 28 to 58 years (two male and two female actors). The video patients represented common mental disorders (depression, anxiety disorder, alcohol addiction, depression after physical disease). The abbreviated names, ages, and disorders of the patients were displayed for 3 s during the video. The patients of the experimental group gave information about the mostly positive outcomes and the processes of their therapy. The same patients acted also in the control group video, providing information about symptoms and the beginning of psychotherapy but not about therapy outcomes. All participants watched a video with four patients (7 min).

Both videos were previously evaluated by 12 experts (psychotherapists and scientists in the area of clinical psychology). The ratings included the following criteria: sympathy, credibility, friendliness, and identification with patients. They also rated the quality of the sound, resolution, length, and size of the video. Because the ratings of the patients’ criteria and the quality of the video were good to very good, we only made small changes.

### Demographic Questions and Therapy Experience

Demographic variables included questions on gender, age, nationality, mother tongue, and educational and vocational qualifications. In the case of existing therapy experience, questions were asked about duration, time elapsed since completion of the last therapy, type of therapy, and therapy outcome (helpful vs. unhelpful). Potential diagnoses and intake of medication were both recorded using one item.

### Primary Outcome

Expectations were captured using a German translation of the Milwaukee Psychotherapy Expectation Questionnaire (MPEQ) (31), adapted in the context of this study (see Supplementary Material). The translation and re-translation were done in cooperation with the authors of the original English version. The content-related correspondence of the items translated into German was checked by a re-translation into English and was confirmed. With a total of 13 items, the MPEQ assesses both process expectations (nine items) and outcome expectations (four items). Answers are given on an eleven-level Likert scale from not at all (0) to highly agree (10).

For the English version, the authors reported good reliability for internal consistency (Cronbach’s Alpha α > 0.85 for both scales) and for retest reliability (2 weeks) with *r* = 0.83 for the process expectation scale and *r* = 0.76 for the outcome expectation scale. In addition, there was evidence for convergent validity [significant correlations with the scales of the Psychotherapy Expectancy Inventory-Revised (32)]. For the process expectation scale, there was also an association with entry into therapy, which can be interpreted as evidence of predictive validity.

### Secondary Outcome

Attitudes towards psychotherapy were recorded with the Questionnaire on Attitudes towards Psychotherapy [QAPT (33, 34)]. With a total of 11 items, this questionnaire records two scales: positive attitudes towards psychotherapy (six items) and acceptance in society (five items). While the positive attitudes towards psychotherapy scale contains statements on the effectiveness of psychotherapy and the competence of the therapist, the acceptance in society scale focuses especially on stigmatization. Answers are made on a four-level Likert scale from do not agree (1) to agree (4). Ditte et al. (33) reported good reliability for a German sample (*N* = 48) with values for Cronbach’s Alpha from α = 0.78 for both scales.

Behavioural intentions were recorded with a total of six self-developed items. The following three facets were assessed with two items each: (1) The intention to inform oneself about psychotherapy, (2) the intention to use psychotherapy for oneself, and (3) the intention to recommend psychotherapy to third parties. The answers were given on a seven-level Likert scale from “no, in no case” (1) to “yes, in any case” (7).

### Covariates

The state of health was recorded using the Brief Symptom Inventory [BSI-18 (35)]. This includes six items each on somatization, depression, and anxiety, which are among the most common mental disorders in the German general population (2). The extent of stress is measured on a five-level Likert scale from not at all (0) to very strong (4). The evaluation is carried out using sum scores, which can be formed both for the single dimensions and for the total value [Global Severity Index (GSI)].

Self-report of perceived sympathy, attractiveness, friendliness, and identification with the patients in the video were recorded using items on a five-level Likert scale.

### Analysis

The statistical evaluation of the data was performed using IBM SPSS Statistics^®^ for Windows, Version 21 (RRID:SCR_019096). For the statistical analysis, the significance level was set at α = 0.05. The data set was checked for missing values. Participants who claimed to know the actors were excluded. Furthermore, fulfilling exclusion criteria and more than one error in the content manipulation check led to exclusion. Subsequently, the descriptive data–such as mean value, standard deviation, and range–were checked for their plausibility and an analysis of very unusual value outliers was carried out.

Pre-tests were carried out to check the equal distribution of demographic and psychosocial characteristics across the two groups. The assumption of normal distribution and homogeneity of variances was checked.

The hypothesis was tested by means of two factor variance analyses (ANOVA) with mixed design. The factor “time” was repeated with two steps (T0, T1) and the factor “condition” was a between-subject factor which also had two steps (control group, intervention group). For the two-factor variance analysis with repeated measurement on one factor, the following assumptions were checked: (1) multivariate normal distribution, and (2) homogeneity of the variances between the levels of the non-repeated factor and homogeneity of the variance-covariance matrices. The multivariate normal distribution was tested approximately over the normal distribution of the dependent variables in the sub-samples. The homogeneity of the variances was checked with the Levene test and the homogeneity of the variance–covariance matrixes was established using the Box’s *M* test.

## Results

**FIGURE 2:**
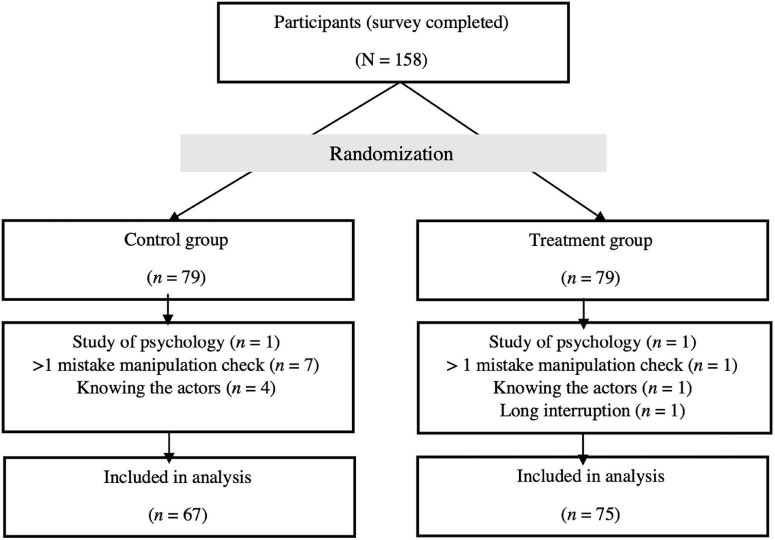
Flow chart of participants.

The total sample of the study consisted of 158 persons. After exclusion of participants, the statistical analysis revealed a sample size of *N* = 142 persons. A participants’ flow chart is shown in Figure 2.

The mean sum score of the Global Severity Index (GSI) of the BSI-18 of Spitzer et al. (35) was *M* = 8.98 (*SD* = 9.03). As expected, this was significantly below the mean value of the clinical sample [*M* = 22.90, *SD* = 14.03 (35)].

The distributions of the sub-samples deviated significantly from the normal distribution on some variables. With reference to the central limit value theorem, the normal distribution of the sample characteristic value distribution can nevertheless be assumed due to the sample size of *n* > 30 (36).

We found a significant difference in the distribution of male and female participants, in favour of more women in the intervention group. Regarding the remaining characteristics, no differences could be found between the sub-samples. For a detailed description of the differences between the sub-samples in demographic and psychosocial variables, see Table 1.

**TABLE 1:** Sample baseline characteristics.

	Control group *n* = 66	Experimental group *n* = 75	
	*M (SD)*	*M (SD)*	Test statistic
Age	29.6 (11.4)	33.6 (14.6)	*t*(139)^a^ = –1.81; *p* = 0.07
Global Severity Index	9.5 (9.6)	8.5 (8.5)	*t*(139)^a^ = 0.66; *p* = 0.51
Sex, *n*	♂: 34	♂: 25	*x*^2^^b^ = 4.42; *p* = 0.04*
	♀: 33	♀: 50	
Education: advanced school-leaving certificate, *n*	56	69	Fisher-Yates: *p* = 0.10
Therapy experience, *n*	19	19	*x*^2^^b^ = 0.17; *p* = 0.71
Therapy experience of a significant other, *n*	58	67	Fisher-Yates: *p* = 0.46

*^a^Independent samples t-test. ^b^Chi-square test of homogeneity.*

* p < 0.05.

**FIGURE 3:**
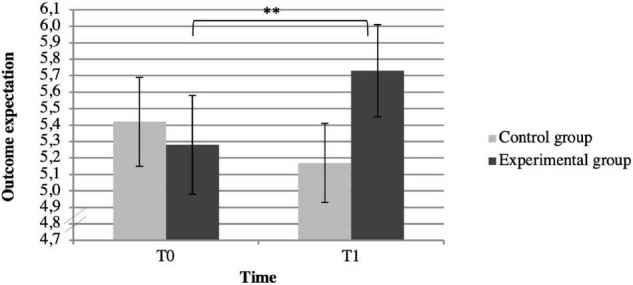
Mean outcome expectation for participants in the control (*n* = 67) and experimental groups (*n* = 75). ^**^ *p* < 0.01.

The results of the two-factor analysis can be found in Table 2. We found a significant interaction between “time” and “condition” for outcome expectation [*F*(1,140) = 9.72, *p* = 0.002, η2 = 0.065]. According to the conventions of Cohen (37), the effect strength of the interaction corresponds to a medium effect. The interaction is shown in Figure 3. The results of the *post-hoc* tests (Table 3) show that the outcome expectation in the intervention group increased significantly (*M*ΔT0, T1 = 0.44, *SE* = 0.15, *p* = 0.002). In the control group, however, there was no significant change in the outcome expectation between the measurements (*M*ΔT0, T1 = –0.22, *SE* = 0.16, *p* = 0.15). The main effect of time [*F*(1,140) = 1.05, *p* = 0.31, η2 = 0.007] and group [*F*(1,140) = 0.31, *p* = 0.58, η2 = 0.002] were not significant.

**TABLE 2:** Two-way analysis of variance for time and condition.

Variable and Source	*df*	*F*	*p*	η ^2^
***Outcome Expectation***				
Condition	1, 140	0.31	0.58	0.002
Time	1, 140	1.05	0.31	0.007
Time × Condition	1, 140	9.72**	0.002	0.065
***Process Expectation***				
Condition	1, 140	0.05	0.83	<0.001
Time	1, 140	35.02***	<0.001	0.200
Time × Condition	1, 140	1.87	0.17	0.013
***Attitudes towards Psychotherapy***				
Condition	1, 140	1.05	0.31	0.007
Time	1, 140	27.59***	<0.001	0.165
Time × Condition	1, 140	3.29	0.07 (*)	0.023
***Acceptance in Society***				
Condition	1, 140	0.11	0.31	0.007
Time	1, 140	10.17**	0.002	0.068
Time × Condition	1, 140	0.77	0.38	0.005
***Behavioural Intentions***				
Condition	1, 140	<0.01	0.96	<0.001
Time	1, 140	19.55***	<0.001	0.123
Time × Condition	1, 140	1.38	0.24	0.010

η^2^ effect size measure of ANOVA. *p < 0.05; **p < 0.01; ***p < 0.001. Significant values in bold.

**TABLE 3:** Descriptive statistics of primary and secondary outcomes for T0 and T1 and results of *post-hoc* tests.

	Control group (*n* = 67)			Intervention group (*n* = 75)		
	*M*(T0) *SD*	*M*(T1) *SD*	*M*(ΔT0, T1) *SE*	*M*(T0) *SD*	*M*(T1) *SD*	*M*(ΔT0, T1) *SE*
Outcome expectation	5.41 (2.16)	5.19 (2.41)	–0.22 (0.16)	5.28 (2.11)	5.73 (2.39)	0.44** (0.15)
Process expectation	6.99 (1.56)	7.27 (1.68)	0.28** (0.09)	6.96 (1.47)	7.40 (1.59)	0.45*** (0.08)
Attitudes towards psychotherapy	18.28 (3.21)	18.88 (3.47)	0.60** (0.25)	18.48 (3.01)	19.71 (2.87)	1.23*** (0.24)
Acceptance in society	12.96 (3.47)	13.61 (3.43)	0.66** (0.24)	12.91 (3.67)	13.28 (3.71)	0.37* (0.22)
Behavioural intentions	5.27 (1.12)	5.44 (1.20)	0.17* (0.08)	5.20 (1.14)	5.49 (1.02)	0.30*** (0.07)

*ΔT0, T1, Change from T0 to T1. Unilateral testing. Range of values for outcome expectations and process expectations from 0 (not at all) to 10 (very). Range of values for attitudes towards psychotherapy and acceptance in society from 0 to 44. Range of values for behavioural intentions each from 1 (no, in no case) to 7 (yes, in any case).*

* p < 0.05; ** p < 0.01; *** p < 0.001. Significant values in bold.

Concerning the other variables (process expectation, attitudes towards psychotherapy, acceptance in society, and behavioural intensions), we found a significant main effect for “time” (Table 2). The secondary outcome measures changed significantly over time both in the control and intervention groups. According to the conventions of Cohen (37), the effect strengths correspond from medium to large effects. The differences in the mean values indicate that the direction of the effect was positive in all cases (see Table 3). Thus, in both groups there was an increase in the values for process expectations, attitudes towards psychotherapy, acceptance of psychotherapy in society, and behavioural intentions. Again, the differences between the mean values were checked for significance with *post-hoc* tests. The increase in mean values was significant for all four secondary outcome measures both in the intervention and control group. The interaction effect of group and time was not significant for any of the other variables. A trend for the interaction of group and time for attitudes towards psychotherapy was found (*p* = 0.07).

## Discussion

The central aim of the present study was to test whether the expectations about psychotherapy of critically minded persons can be improved with the help of an expectation-optimizing video. We were able to show that outcome expectations improved more substantially in participants of the experimental group compared with participants of the control group at the level of a medium effect, according to Cohen (37). Therefore, seeing a video with patients talking about their positive therapy experiences is more effective for changing therapy outcome expectations than just seeing a video with patients talking about their symptoms. Unfortunately we couldn’t find a difference between the two videos for attitudes towards psychotherapy and intentions to seek psychotherapy as these variables were increasing for both groups over time.

This is consistent with the results of various studies that have also raised expectations or related variables such as attitudes and intentions (19–21, 25, 28, 38–40). In contrast with the present study, however, many of the cited works addressed and changed other facets of expectations. These include, for example, control expectations (39), expectations regarding the length of treatment (40), or the acceptance of treatment (19, 38). The results show greater methodical parallels with the studies by van Osch et al. (21) and by Kazdin and Krouse (20). As in the two studies just mentioned, the outcome expectation in the present study could be increased by specifically mentioning positive treatment aspects or successful treatment histories. Thus, the present study agrees with the position that reports of a positive treatment result can lead to an increase in outcome expectations. As van Osch et al. (21) and Tinsley et al. (27) are recommending, video material is suitable as a medium for manipulating expectations. This study also supports that observational learning is a mechanism by which changes in expectations can be achieved. This is in line with the results of Kazdin and Krouse (20), who also used positive patient reports as expectation manipulation. In the study by van Osch et al. (21) it is also feasible that the viewers’ identification with the patient shown in the video played a considerable role in the changes in expectations. The extent of identification with the patients shown in the video was determined in the present study in the form of a self-report. The underlying mechanisms of a successful manipulation of expectations should be examined in more detail in future research.

Although the postulated differential effect between the intervention group and the control group became apparent for the primary outcome measure of outcome expectation, for all other (secondary) outcome measures, only an increase of the measures in both conditions could be detected, whereas at least the interaction of group and time for attitudes towards psychotherapy resulted in *p* = 0.07. The difference in the result pattern between the primary outcome and the secondary outcome measures might be related to the fact that the changes were based on different mechanisms of action. In the conception of the intervention video, special attention was paid to the expected outcomes. The main distinguishing feature of the videos was that the results of psychotherapy were only mentioned in the reports of the intervention video but remained open in the control video. This could have been decisive for the differential effects between the videos. The exposure to mentally ill people was similar between both videos and, therefore, according to the contact theory of social psychology (29, 41), similar effects were achieved for variables such as process expectations, acceptance of psychotherapy in society, and behavioural intentions. Various studies have shown that contact with mentally ill persons can lead to the reduction of stigma, as mentioned before.

## Strengths and Limitations

This work used self-produced videos in which the roles of fictive patients were played by actors. By using the same fictitious patients, framework information, and video length, a high level of standardization and comparability of the control and intervention videos could be achieved. Furthermore, various quality criteria of the videos were evaluated in pre-tests and were rated as being good.

A high-quality feature of the study was that key variables (such as the primary outcome measure of outcome expectations) were collected using standardised measurement instruments. These were proven to be valid and reliable in previous studies. The MPEQ (31) was translated into German for the first time for the present study, and the content-wise consistency of the two versions was confirmed by a translation and a re-translation. The translated version also proved to be reliable in the present sample with values for Cronbach’s Alpha at α = 0.79 for the scale process expectations and α = 0.78 for the scale outcome expectations. In the absence of standardised instruments for other variables, self-created scales were used. There are no data on reliability and validity for these and they should therefore be interpreted with caution.

One of the advantages of the online study approach is that the absence of a test leader did not lead to any bias due to test leader effects (42). On the other hand, one disadvantage of the used online setting is that there is not much control over the study environment (43). Neither the identity of the participants can be verified, nor can the behaviour of the participants be observed. Even external disturbing factors cannot be controlled. Therefore, a manipulation check was included which made it possible to ensure, as far as possible, that the participants had watched the video with a certain amount of attention.

Another strength of the experimental design was the randomised (and blind) allocation of the experimental conditions. Consequently, selection effects within the sample can be excluded. The participants of both groups were truthfully informed in the study information that the aim of the study was to investigate whether and how expectations of psychotherapy could be improved. Although this transparency is particularly advantageous from an ethical perspective, it could have strengthened the demand characteristics of the study. However, the different results of the control and intervention groups with respect to the outcome expectations, on the one hand, and equality with respect to the other outcome measures, on the other hand, make a distortion of the result pattern by demand characteristics appear unlikely.

Additionally, integrating follow-up measurements into future studies could provide insights into the long-term effects of expectation manipulation. Results to date are valid regarding a sample that is critical towards psychotherapy. Further studies should focus on other samples as well.

## Conclusion

The present study is one of the first studies that has investigated the effects of expectation manipulation on changes in expectations under experimental conditions. The results support findings that it is possible to positively influence treatment outcome expectations. Within the scope of the present study, a positive effect on the outcome expectations of psychotherapy in a mainly healthy sample could be achieved by using a video with patient reports. Due to the positive association between favourable outcome expectations and the use and success of psychotherapy, this has promising implications for prevention programs. The use of expectation-optimizing videos could be used, for example, in the context of educational campaigns. Videos such as the one produced for this study are an economical way of conveying information. They could help to improve attitudes and expectations towards psychotherapy in the general population. Also, in the psychotherapeutic setting, expectation-optimizing videos could be used to increase the treatment expectations of patients. Checking the effectiveness of expectation-optimizing videos within a patient sample could be an important next step in research.

## Data Availability Statement

The datasets generated and analyzed during the current study are not publicly available due to data privacy (heath data) but are available from the corresponding author on reasonable request.

## Ethics Statement

The studies involving human participants were reviewed and approved by Ethics Committee of the Psychological Department, University of Marburg. The participants provided their written informed consent to participate in this study.

## Author Contributions

Both authors designed the study, made substantial contributions to the conception of the work, and approved the final manuscript. KB-K additionally was responsible for the acquisition and analysis of the data.

## Conflict of Interest

The authors declare that the research was conducted in the absence of any commercial or financial relationships that could be construed as a potential conflict of interest.

## Publisher’s Note

All claims expressed in this article are solely those of the authors and do not necessarily represent those of their affiliated organizations, or those of the publisher, the editors and the reviewers. Any product that may be evaluated in this article, or claim that may be made by its manufacturer, is not guaranteed or endorsed by the publisher.
