# Supplementary material for: Maintenance vs. Change of Negative Therapy Expectation: An Experimental Investigation Using Video Samples
Source: Front Psychiatry. 2022 Apr 4;13:836227. doi: 10.3389/fpsyt.2022.836227 (PMC9013760; doi:10.3389/fpsyt.2022.836227)
Supplement: Supplementary file 1 [file Data_Sheet_1.DOCX]

Supplementary Material

**Adaption to the instruction of the MPEQ** (accepted by original authors)

Now we would like to ask you to imagine that you are experiencing a high amount of (mental) distress, and that life is not going the way you wished it would be. If you are currently experiencing a high level of distress, then you do not need to imagine, just focus on how you feel right now. Imagine furthermore that you are thinking about contacting a psychotherapist because of this distress and dissatisfaction. Please answer the following questions concerning expectations on psychotherapy from this perspective. Thank you.

**Script of control video**

*Pat 1: Female; age between 20-30 years, student, alcohol addiction*

`I started going to psychotherapy last week. I have yet to tell my friends and family about it and I am honestly really scared of their reaction. I couldn't admit to myself for a long time that I was no longer in control of my alcohol consumption. All my friends are partying a lot and are drinking alcohol. At some point, alcohol became more than just a party drink for me. I started drinking alone in the evenings during the week, at first only 1-2 glasses of wine, then at some point I was up to a bottle. Later I started drinking at lunchtime. At some point, this also influenced my studies. I regularly miss seminars because I'm hungover in the morning and when I do manage to get up, I can't concentrate.

My therapist explained to me how addiction develops and what the physical effects of it are. My therapist said that one or two relapses are part of the disease. I guess we look closely at typical relapse situations in therapy and think about what makes them so difficult, where I can still intervene and what I would do differently next time. `

*Pat 2: Male, age between 30-40 years, construction worker*

`I have recently been diagnosed with depression by my doctor. He first informed me about typical symptoms. He mentioned, for example, dejection, lack of drive and social withdrawal. This reminds me a lot of myself. For example, I was a member of a sports club for a long time, but at some point, I stopped going to training because it was no use anyway. Or last week there was this situation: my colleagues asked me if I wanted to go out for dinner with them after work. But I just want to go home and don´t see anyone. I already have enough problems of my own. In general, I don't really feel like doing anything anymore, I would prefer to just lie in bed and be left alone by everyone.

My doctor has now advised me to have psychotherapy because of my depression and has informed me about different forms of psychotherapy. I'm looking forward to it, after all we're not talking about a new problem I have. Last week I had my first session, and we had the opportunity to talk a bit about the therapy process: We are going to analyze my behavior in everyday life and with others. Weekly plans are also set up, which are increased every week. I have another appointment next week. We'll see what we do there. `

*Pat 3: Female, age between 40-50 years, housewife, social anxiety disorder*

`It´s hard for me to talk about my anxiety disorder. My therapist diagnosed a social phobia. This manifests itself in the fact that I almost completely avoid activities with other people and in public. However, going into such situations causes me to feel extremely strong anxiety and discomfort. I worry a lot about others seeing my anxiety symptoms, i.e. sweating, blushing and trembling, and that they could laugh about me.

I had my first anxiety attack probably 20 years ago when I was supposed to train new trainees at work. I couldn't get a word out, I was blushing and just left the room. Later I told the others that I just wasn't feeling well. I had the feeling that they didn't believe me. After that I have stayed at home more often and quit my job.

At first, I assumed that my therapist would only talk to me about the anxiety or give me a pill against the symptoms. He then explained to me that exercises in which I face my fears are a crucial part of the therapy. I was very surprised that I am supposed to give presentations in front of others and have to practice at home. I am honestly quite afraid to face the group exercises. I really don't trust myself to do that. I'm curious to see how it goes. `

*Pat 4: Male, age between 50-60 years (retired), cancer diagnosis 2 years ago*

`When I was diagnosed with cancer 2 years ago, it was an absolute shock. Not only for me but also for my whole family. I was incredibly scared. I thought my life was over. I hardly said anything to my wife because I didn't want to put any more stress on her. An operation followed, during which the tumor was removed, then 6 months of chemotherapy. So far, the follow-up check-ups have all been good, but still, nothing in my life is the same as before. My body is completely worn out from the after-effects of the chemo. I am constantly exhausted, no matter how much I sleep. My body is like slowed down and I'm only running at 50%. Besides, there's no way to just carry on as before, while my wife just wants to get back to normal. Additionally, there is this constant worry of a relapse. My wife recently made an appointment for me at the cancer counselling center, who referred me to a psychotherapist. I wanted to do anything but starting a treatment again, but then I made an appointment. I had my first appointment now and it was quite difficult for me to tell the therapist about myself. I mean, I hardly know anything about him, even though it's quite relieving that the therapist is bound by confidentiality. `

**Script of experimental video**

*Pat 1: Female; age between 20-30 years, student, alcohol addiction*

´I have been in psychotherapeutic treatment for my alcohol addiction for about a year. I hid it for a long time because, to be honest, I was afraid of the reaction of my friends and family. But when I did talk about it once, I was amazed at how many others had similar experiences. I was also afraid of being judged by my therapist at the beginning. Surprisingly, my therapist was very understanding. She explained to me how addiction develops, what the physical effects of addiction are and how difficult it is to stop on your own and break out of this vicious circle.

In the process, I noticed how I built up more and more self-worth. I managed to stay sober for months. At some point, however, I relapsed. I was sure that all the work I invested had been useless. In therapy, we took a closer look at the relapse situation and thought about what had made it so difficult for me, where I could have intervened and what I would do differently next time. I found that quite helpful. My therapist said that one or two relapses are part of the disease and that I should not give up hope. It helped me that she continued to support me and didn't lose faith in me. So far, there have been no further relapses and I feel more stable. My academic performance has also improved a bit. I almost miss no seminar and my concentration is much better. `

*Pat 2: Male, age between 30-40 years, construction worker, depression*

`My doctor advised me to have psychotherapy about half a year ago because of depression. To be honest, I didn't think there was much chance of success at the beginning. After all, I had known about my problem for years.

But the therapy went differently than I thought. Surprisingly, one or two things did change. We took a close look at my behavior in everyday life and with others. It sounds a bit stupid, but I gathered a better understanding of myself. My therapist wasn't always `nice` to me and sometimes questioned my judgements of a situation or a thought. In the end, there was no magical change at one point. I was hard work for me, we set up weekly activity plans and added new activities every week. I eventually became more active again. I was a member of a sports club for a long time, but at some point, I stopped going to training because I thought it was all useless anyway. Sport and the people I meet there are now something I look forward to again. I also get much better along with my colleagues now. Before therapy, I just wanted to go home after work and don`t see anyone. I already had enough problems of my own. My therapist discussed possible fears and worries with me and encouraged me to approach others more openly again. Before therapy this was unimaginable for me. `

*Pat 3: Female, age between 40-50 years, housewife, social anxiety disorder*

`It´s hard for me to talk about my anxiety disorder. My therapist diagnosed a social phobia. This manifests itself in the fact that I almost completely avoid activities with other people and in public. However, going into such situations causes me to feel extremely strong anxiety and discomfort. I worry a lot about others seeing my anxiety symptoms, i.e. sweating, blushing and trembling, and that they could laugh about me.

I´ve done a therapy before, but my anxiety didn´t change. I didn't feel that comfortable with the therapist and didn't really want to get involved with the exercises. It took a lot of effort to talk it over with the therapist, but he was understanding and helped me to find another therapist. My second therapy was exhausting but very helpful.

Initially, I was totally afraid to do group exercises. At first, I assumed that my therapist would only talk to me about the anxiety or give me a pill against the symptoms. I was very surprised that I would have to give presentations in front of others. I would have never dared to do that. I then continued to practice these things at home. At the end of the therapy, I had the feeling that I was much better in coping with the anxiety. I now dare to take the bus and go shopping again, which would have been unimaginable for me at the beginning of therapy. My family has also told me that I am happier and more adventurous again. I am proud of what I have achieved so far. `

*Pat 4: Male, age between 50-60 years (retired), cancer diagnosis 2 years ago*

`When I was diagnosed with cancer 2 years ago, it was an absolute shock. I was incredibly scared. I thought my life was over. I hardly said anything to my wife because I didn't want to put any more stress on her. I had an operation to remove the tumor, then 6 months of chemo. So far, the follow-up check-ups have been all good, but still, nothing in my life was the same as before and I didn't want to pretend that it was. At the same time, my wife just wanted to get back to normal after the long and exhausting treatment. That was quite difficult for me. In addition, there was the constant thought in my head that there could always be a relapse.

Through the cancer counselling center, I was then referred to a psychotherapist. At first it was very difficult for me to tell the therapist about myself and my fears. I mean, I hardly knew anything about him. Then I was quite relieved that the therapist was bound by confidentiality, and I additionally felt that I wasn't under any pressure in the sessions. Over time, I found it easier to be open about my feelings and it felt good to sort out my thoughts and fears with someone else. I could talk openly about my fears of relapse without feeling like I was burdening my therapist. Meanwhile, almost a year later, I know that my life is not going to be the same as before, but I have regained perspective on life and worked out new goals. We have also discussed how I can speak more openly with my wife again. We now talk a lot more to each other and have goals and plans for our life. `
